# Cultural competence of mental health professionals

**DOI:** 10.1192/j.eurpsy.2022.1402

**Published:** 2022-09-01

**Authors:** A. Zartaloudi

**Affiliations:** University of West Attica, Nursing, Athens, Greece

**Keywords:** immigrants, Cultural competence, mental health professionals

## Abstract

**Introduction:**

Societies nowadays, including Greece, are usually multicultural. Health professionals should therefore be properly trained to consider patients’ beliefs, attitudes and particular needs depending on their different cultural background.

**Objectives:**

To identify the features that the culturally competent professional should have in order to understand better the nature of cultural competence and its importance to mental health professionals in early intervention of immigrants’ mental health problems.

**Methods:**

A literature review has been made through PubMed database.

**Results:**

The development of cultural competence is a continuous process. Culturally competent professionals should have the following features: a) Understand the concept of culture and the way individuals’ cultural background affect their feelings and their intercultural interactions. b) Choose appropriate collaboration strategies with people from different cultural backgrounds. c) Accept diversity and respect patients’ differences, demands and choices without criticism while providing them the proper care. d) Be fair and take care of all patients without any distinction regardless of the language they speak. e) Familiarize themselves with issues related to mental health and illness and encourage patients to explain how their illness affects their lives. Culturally competent mental healthcare professionals should seek more than the provision of caring without prejudice. They should respect the positive contribution of cultural origin and identity to people’s well-being, learn their life stories and develop a relationship of trust with each patient separately.

**Conclusions:**

Cultural competence might help mental health professionals to understand and provide adequate services with respect to patients who come from a different cultural background.

**Disclosure:**

No significant relationships.

